# Effects of foot and ankle mobilisations combined with home stretches in people with diabetic peripheral neuropathy: a proof-of-concept RCT

**DOI:** 10.1186/s13047-023-00690-4

**Published:** 2023-12-06

**Authors:** Vasileios Lepesis, Joanne Paton, Alec Rickard, Jos M. Latour, Jonathan Marsden

**Affiliations:** 1 School of Health Professions, Faculty of Health, University of Plymouth, Plymouth, UK; 2 School of Nursing and Midwifery, Faculty of Health, University of Plymouth, Plymouth, UK

**Keywords:** Mobilisations, Diabetes, Neuropathy, Limited joint mobility syndrome, Peak planar pressures

## Abstract

**Introduction:**

People with diabetic peripheral neuropathy (DPN) and limited joint mobility syndrome (LJMS) can experience increased forefoot peak plantar pressures (PPPs), a known risk factor for ulceration. The aim of this study was to investigate whether ankle and 1st metatarsophalangeal (MTP) joint mobilisations and home-based stretches in people with DPN improve joint range of motion (ROM) and reduce forefoot PPPs.

**Design and methods:**

Sixty-one people with DPN (IWGDF risk 2), were randomly assigned to a 6-week programme of ankle and 1^st^ MTP joint mobilisations (*n* = 31) and home-based stretches or standard care only (*n* = 30). At baseline (T0); 6-week post intervention (T1) and at 3 months follow-up (T2), a blinded assessor recorded dynamic ankle dorsiflexion range using 3D (Codamotion) motion analysis and the weight bearing lunge test, static 1st MTP joint dorsiflexion ROM, dynamic plantar pressure and balance.

**Results:**

At T1 and T2 there was no difference between both groups in ankle dorsiflexion in stance phase, plantar pressure and balance. Compared to the control group, the intervention group showed a statistically significant increase in static ankle dorsiflexion range (Left 1.52 cm and 2.9cms, Right 1.62 cm and 2.7 cm) at 6 (T1) and 18 weeks (T2) respectively *p* < 0.01). Between group differences were also seen in left hallux dorsiflexion (2.75°, *p* < 0.05) at T1 and in right hallux dorsiflexion ROM (4.9°, *p* < 0.01) at T2 follow up. Further, functional reach showed a significant increase in the intervention group (T1 = 3.13 cm *p* < 0.05 and T2 = 3.9 cm *p* < 0.01). Intervention adherence was high (80%).

**Conclusions:**

Combining ankle and 1^st^ MTP joint mobilisations with home-based stretches in a 6-week programme in people with DPN is effective in increasing static measures of range. This intervention may be useful for improving ankle, hallux joint mobility and anteroposterior stability limits in people with diabetes and neuropathy but not for reducing PPP or foot ulcer risk.

**Trial registration:**

https://classic.clinicaltrials.gov/ct2/show/NCT03195855.

**Supplementary Information:**

The online version contains supplementary material available at 10.1186/s13047-023-00690-4.

## Background

In 2019, the International Diabetes Federation estimated that 463 million adults were living with diabetes; this number is predicted to rise to 700 million by 2045 [1]. The healthcare cost of treating diabetic foot disease, including diabetic foot ulceration (DFU), in the United Kingdom also set to rise to 15.1 billion pounds by 2035 [2]. The 5-year mortality rate for DFU is between 43%-55% [3]. Whilst managing the risk of DFU poses one of the biggest challenges faced by healthcare professionals involved in diabetic foot care due to its highly complex multifactorial aetiology and pathogenesis [4]. Part of the multi-aetiology for DFU in the forefoot remains the exposure of insensate deformed feet to high peak plantar pressures (PPPs) [5]. The defined optimal cut-off point of PPP threshold to predict risk of DFU seems to be elusive [6], with one study reporting this critical level at 700 kPa [7]. Another dominant factor in the causation of increased PPP and subsequent DFU development is limited joint mobility syndrome (LJMS) [8]. Biochemically, LJMS is thought to occur due to the accumulation of non-enzymatic glycosylation of collagen with the formation of advanced glycation end-products [9]. Increases in collagen cross-linking alters the mechanical properties of joints and tendons, leading to decreases in elasticity [10]. This manifests with stiffness and reduction in range of motion (ROM) of the ankle and 1^st^ metatarsophalangeal joint (MTPJ) [11] and alterations in normal foot rollover during gait [12].

Stiffness in the ankle and 1^st^ MTPJ lead to the disruption of the functional rockers of gait. These rockers aim to facilitate a wheel-like rolling motion under the foot in the sagittal plane of motion and help minimise the vertical oscillations of the centre of mass (CoM) [13]. It has been suggested that the restriction in ankle range results in an abrupt end-point in forward progression in the late ankle rocker phase [14]. When there is not enough ankle dorsiflexion, the heel lifts prematurely from the ground, elevating the CoM [15]. Based on Newton’s third law, the forces associated with elevating the centre of mass push the metatarsal heads into the ground [15], resulting in increased ground reaction forces and plantar pressures [16]. Biomechanical compensations are thought to include early heel lift and prolonged forefoot loading patterns [17]. This theory is also proven by studies in people with diabetes who presented with restriction in ankle ROM and underwent surgical lengthening of their Achilles tendon. The procedure was successful in increasing ankle ROM which resulted in reductions of PPPs around the forefoot [18–22]. Therefore, if foot and ankle dorsiflexion (DF) ROM can be re-established, then a more normal foot rollover gait may be restored, and PPP and associated DFU risk reduced.

Other than the risk of DFU, people with diabetes tend to have impaired balance and increased risk of falling [23]. The maintenance of functional balance relies on the central integration of afferent information from visual, vestibular and somatosensory systems [24]. In people with diabetes and neuropathy, the loss of cutaneous sensory receptors in the plantar surface of the foot [25] and LJMS [26] seem to be associated with altered awareness of lower limb positioning and functional restriction at the ankle, leading to reduced postural stability. When people with diabetes and neuropathy detect postural instability, they tend to adopt a more rigid posture involving muscle co-contraction to aid stability [27]. However, increased ankle joint DF may improve mechanical stability and stimulate mechanoreceptor activity to enhance the proprioceptors within the joint which aid balance.

Manual therapy (passive movement applied to the joint) aims to restore optimal ROM and function by re-establishing the arthrokinematic accessory gliding and rolling movement that is associated with normal joint movement [28]. The effectiveness of these techniques in increasing ankle DF ROM and improving balance have been explored in previous studies of people with chronic ankle instability [29–31]. However, until now, foot and ankle mobilisations have not been applied to people with diabetes and LJMS [32].

Stretching exercises, are often prescribed as an adjunct to manual therapy by addressing limitations of joint ROM which have a contractile (i.e., muscle) component as the source of limitation [33]. Increase in passive muscle tension and joint stiffness, secondary to long-term exposure of soft tissues to non-enzymatic glycosylation changes have been reported in the literature [34]. The aim of stretching is to increase the resting muscle length, thereby increase joint ROM [35]. The literature supporting the use of stretches in people with diabetes and ankle limitation ROM is inconclusive; some studies report an increase in ankle ROM [36, 37] whilst others found no change [38, 39]. A recent review also highlighted this gap and the need for future research on the effects of specific foot and ankle exercise components on people with diabetes [40]. This inconsistency in treatment effect might be attributed to poor adherence to exercise, estimated to reach levels of 30% to 53% [41]. Whereas it is thought that minimal acceptable adhesion levels should be as high as 80% to 85% for intervention results to be effective [42].

The aim of this study was therefore to compare the effect of a bilateral 6-week ankle and 1^st^ MTPJ mobilisation intervention plus a home-based stretching programme, to usual foot care, on ankle and 1^st^ MTPJ DF ROM, PPPs, balance and adherence to exercise in people with diabetic peripheral neuropathy (DPN).

## Methods

A single site, parallel group, two-arm, proof of concept (PoC) assessor blinded randomised controlled trial (RCT) was adopted. Reporting followed CONSORT Guidance [43]. The trial received approval from the HRA Southwest – Exeter Research Ethics Committee (REC: 17/SW/0170), University of Plymouth Research Ethics Committee (17/18–866), and Livewell Southwest R&D department. Informed consent was obtained from all participants prior to enrolment.

Sample size calculation was based on a previous RCT of exercises (including ankle stretches) in people with DPN [39] that found a significant improvement in ankle dorsiflexion (effect size = 1). Taking a conservative approach that we would obtain an effect size of 0.8, a sample size of 26/group (α = 0.05, power = 0.8) was required. To account for a 10%, drop out we aimed to recruit 58 people in total (29 per group).

People with DPN were recruited from two local podiatry clinics between 22^nd^ of May 2018 to 3^rd^ of April 2019. Potential volunteers were initially screened by telephone interview to determine if they met the following criteria: self-reporting DM diagnosis and loss of sensation in feet, ability to walk 10 m independently with or without walking aid and intent to attend 6 sessions over a 6-week period. Exclusion criteria were an existing foot ulceration, a diagnosis of ankle osteoarthritis, rheumatoid arthritis or osteoporosis, a history of a recent fracture or surgery, lower limb amputation or additional neurological or oncological affecting the legs. Final screening was clinically determined prior to obtaining written informed consent. Clinical inclusion criteria were a moderate risk of foot ulceration (International Working Group on the Diabetic Foot (IWGDF) risk 2) [44], ankle and hallux joint stiffness (defined as 0° or less ankle dorsiflexion and < 10° hallux dorsiflexion). Participants were excluded if they presented with excessive distal lower limb oedema preventing mobilisation rated as > 0.6 cm indentation to finger pressure and an inability to palpate the joint line or Charcot arthropathy.

An independent assessor (JM) managed the computer generated randomisation schedule, (Minim – www-users.york.ac.uk) minimised for age (< 70 vs ≥ 70 years) [45]. Age was used as a covariate since glycosylation and joint stiffness is frequently observed in elderly people [46], and these complications are accelerated in people with diabetes [47]. Thus, we aimed to minimise the additional imbalance potentially caused by ageing and the associated effects on joint stiffness. Following informed consent, participants were randomised. Participants in the intervention group attended further appointments to receive the intervention from the physiotherapist (AR). All other members of the research team were blind to the treatment allocation.

### Intervention and usual care

The intervention group underwent bilateral ankle and 1^st^ MTP joint mobilisations (once a week for 6 weeks) and a 6-week home-based programme of stretching exercises. Mobilisations were delivered by a trained physiotherapist with the participant lying in supine and the heel at the edge of the plinth. These consisted of two, 2-min sets of grade II joint traction followed by 2-min sets of Maitland grade III anterior-to-posterior talocrural mobilisations with one minute rest in between sets. The traction was operationally defined as grade II distraction between the joint surfaces, applied intermittently to the point of feeling an increase in the joint space and prior to tissue resistance [48]. The mobilisation was operationally defined as grade III, 1 sec large amplitude rhythmic oscillations, performed into firm resistance or up to the limit of the available range [49] Two sets were performed for each ankle and one for each 1^st^ MTP. The direction of mobilisation force was parallel to the treatment plane and perpendicular to the treatment plane during traction [30]. Home-based stretches were taught in session 1 (Additional File 1) and outlined in an accompanying booklet that also contained a weekly exercise diary (Additional File 2). Stretching technique was visually checked weekly by the physiotherapist. Stretches targeted gastrocnemius, soleus and plantar fascia with a recommended two consecutive static stretches for 20-30 s per day.

Usual care included regular monitoring of foot health by podiatrists as indicated by IWFDF [44] guidelines. A review of current clinical practice within the podiatry clinic indicated that people with moderate/intermediate risk were reviewed every 3 months. Interventions included neurovascular assessment and monitoring, nail care, callus debridement and provision of footwear and specialist insoles.

### Baseline laboratory assessment

Demographic details recorded at baseline (T0) include self-reported duration and type of diabetes, age, gender, weight, ulcer history and retrospective falls rate over the previous 3 months.

Clinical risk factors for diabetic foot ulceration were recorded including presence of LJMS prayer [50] and table top signs [51], Foot Posture Index-6 (FPI) [52], Forefoot deformity score [53], visual presence of Forefoot and pinch callus, neuropathy severity as determined by number of correct responses out of 10 using a 10 g monofilament and 1^st^ MTP ascending vibration thresholds [54], presence of peripheral arterial disease determined by palpation of the dorsalis pedis artery and graded on a 0–4 scale.

Participants attended the Human Movement and Function Laboratory at Peninsula Allied Health Care, University of Plymouth on three separate occasions to complete collection of outcomes measures at a) baseline assessment (T0), b) at the end of the 6-week intervention period or immediately after 6-weeks (T1) and c) at 18-weeks follow-up period (T2) from the baseline. All outcome measures were taken by an assessor (VL) blinded to the group allocation.

### Primary outcome measure

**Fig. 1:**
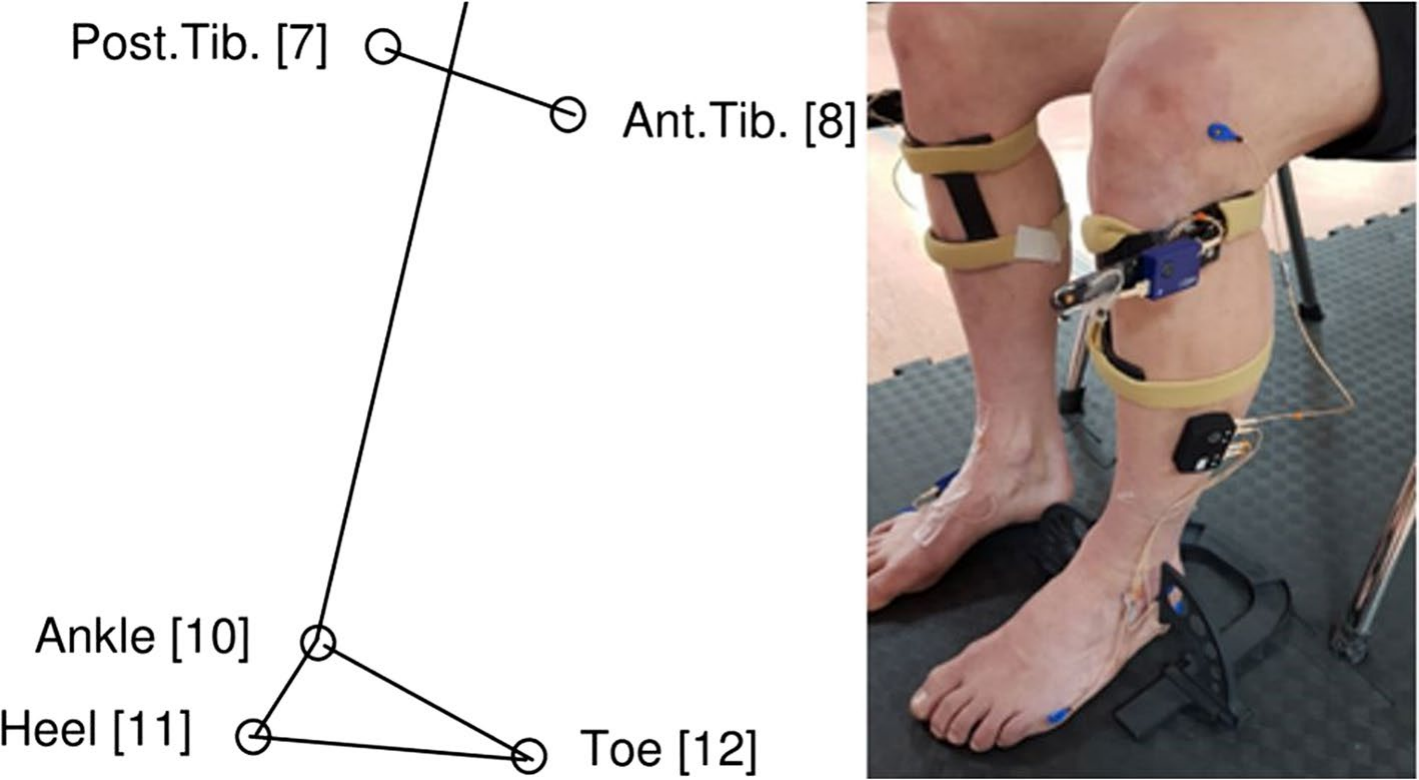
Position of the reflective markers on the lower leg. Reflective markers on the **a** lateral joint line of the knee (9), **b** on the wand (posterior tibia (7) and anterior tibia (8)), **c** prominence of the lateral malleoli (10), **d** lateral border of calcaneum (11) and **e** lateral prominence of the 5^th^ MTPJ (12). Adapted with permission from Coda CX1 user guide. (Charnwood Dynamics Limited [55], p.62)

The primary outcome was ankle dorsiflexion in gait. Amplitude of ankle dorsiflexion during stance phase measured using the Cartesian Optoelectronic Dynamic Anthropometer (CODA) motion analysis (Charnwood Dynamics Ltd., Leicestershire, UK). Markers were placed on the lower leg in standardised positions [55]. In total, six markers were used per right and left side. These were placed on the lateral joint of the knee, on the wand (posterior tibia and anterior tibia), prominence of the lateral malleoli, lateral aspect of the calcaneum and the lateral prominence of the 5^th^ MTPJ (Fig. 1). The Codamotion software was used to analyse the foot and ankle ROM from the sensor modules. The arrays of markers placed on the legs determined a local coordinate system or embedded vector basis (EVB) for each segment. The EVB consisted of three orthogonal axes (Table 1) from which unit vectors were defined. These were used in the measurement of segmental rotations [55].

**Table 1:** Derivation of embedded vector basis (EVBs) for each segment

**EVB**	**Principal axis**	**2**^**nd**^** axis**	**3**^**rd**^** axis**
Foot	Line connecting the heel and toe markers that is offset by ½ inter-malleolar distance (u_x_)	Line running from the heel marker to ankle marker and orthogonal to the principal axis (u_z_)	Orthogonal to 1^st^ and 2^nd^ axes (u_y_)
Shank	Ankle joint centre to knee joint centre (u_z_)	Tibial wand orientation orthogonal to principal axis (u_x_)	Orthogonal to 1^st^ and 2^nd^ axes (u_y_)

The change in the left and right ankles were analysed separately and were considered independent of each other, as supported by studies in healthy adults who show differences between sides during weight bearing ankle DF ROM measurements [56, 57]. In addition, no pooling of right and left limb data was considered for statistical analysis to double the sample size. A calibration trial with the foot at 0° ankle dorsiflexion was taken in sitting (Fig. 1). Participants walked barefoot at their preferred speed along a 10-m walkway with space at each end to allow for gait acceleration and deceleration. Joint angles were calculated using software packages (CODAmotion, Charnwood Dynamics Ltd., Leicestershire, UK) and exported for secondary analysis in MATLAB™. Initial foot contact and foot lift off were identified via the kinematic data using a foot velocity algorithm [58]. The foot velocity algorithm consisted of low pass filtering (7 Hz 4^th^ order zero-phase Butterworth filter) the heel and toe data. The midpoint between the heel and toe markers was then calculated; termed the foot centre. The first derivative of the vertical coordinates of the foot centre was then calculated to give the vertical foot velocity. Characteristic troughs and peaks indicating foot contact and lift off were then identified visually [58] and marked using crosshairs.

A mean of five steps captured from an average of three walking trials were used for data analysis.

### Secondary outcome measures

**Fig. 2:**
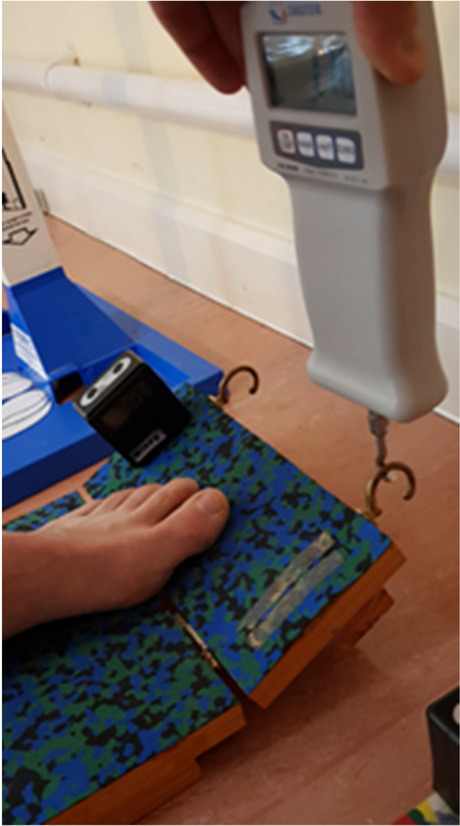
Hallux dorsiflexion measuring rig. The participant was instructed to position their foot in a way that the 1^st^ MTPJ lay over hinge. The researcher then lifted the distal segment of the rig which was attached on a strain gauze. The reading was taken with a digital inclinometer previously calibrated to 0 degrees

The following secondary outcome measures were recorded:

- Stride length: recorded on the walkway as the distance between the heel markers using CODA analysis.
- Forefoot PPP: pressure was measured barefoot using a two-step gait initiation protocol [59, 60] using a pressure sensing mat (Tekscan MatScan system, Tekscan Inc., Boston, USA), calibrated to bodyweight. On average, eight to 10 trials were carried out to capture three steps for each foot. A mean forefoot peak pressure value of the three steps was calculated (ICC = 0.75) [61]. Inaccurate trials were excluded, for example if the participant targeted, missed, stopped on the mat.
- Forefoot-to-rearfoot pressure ratio (F/R ratio): using FootMat software the forefoot (F) and (R) rearfoot regions were masked. The forefoot-to-rearfoot pressure ratio value was derived from the foot with the highest pressure using the formula: F/R. Increases in the F/R ratio are thought to play a prominent role in DFU [62].
- Postural Sway: unperturbed balance was assessed during a 30-s quiet standing period using a portable force plate (Kistler). The centre of pressure (antero-posterior and medio-lateral) velocity was calculated as participants stood with arms by their side and barefoot, and their feet parallel and 4 cm apart with eyes open and closed. The order of testing was randomised by the assessor. Three readings per condition were taken and the mean value was calculated. If participants were required to step or hold onto a support during the trial, another trial was recorded following the participant’s consent. Force plate data was filtered (Butterworth low pass (20 Hz, 1st order) offline and the centre of pressure velocity calculated.
- Functional Reach test (FRT): participants stood in front of a graph paper grid with the arm outstretched and the hand in a fist [63]. The participant was instructed to reach forwards without taking a step and the horizontal distance moved by the 3rd metacarpal head was measured. A familiarization trial followed three readings and the mean value of these recordings was calculated.
- Bilateral static ankle joint dorsiflexion: the weight-bearing lunge test was used to assess static ankle dorsiflexion ROM on both feet [64]. This test was carried out against a stable surface (i.e., door frame). Participants were asked to stand with both feet facing the wall. Participants were then instructed to lunge forward until their knee touched the door frame without lifting their heel off the floor. Participants were given 5 attempts to achieve the greatest distance between their big toe and the wall, for each ankle. Attention was paid to avoid midfoot dorsiflexion [65]. The maximum distance in centimetres (cm) from the wall to the tip of the big toe was recorded using a tape measure positioned on the floor.
- Bilateral static hallux dorsiflexion: weight-bearing static hallux dorsiflexion ROM was measured using a rig consisting of two segments of wood that were hinged [66]. The subject was asked to stand on the rigs with the centre of the 1st MTP joint positioned over the hinge in their natural base of stance whilst looking straight ahead. The distal segment of the wood was then hinged upwards via a connected in-series strain gauge until resistance was felt whilst or when a maximum force of 2 Kgs was applied. The hallux dorsiflexion angle was measured via a digital inclinometer attached to the distal segment of the rig [66] (Fig. 2). A familiarisation trial was carried out, before an average of 3 readings per hallux were recorded.
- Walking scale-12: this is a 12-item questionnaire of self-perceived walking ability [67]. In our study it was used to measure the impact of diabetes on our participants’ perceived walking ability and it is a validated measure of walking and lower limb function [68].
- Exercise adherence and fidelity: the number of therapy sessions attended in the intervention group and the intervention delivered was recorded from standardised therapy notes (Additional File 3). The number of exercises performed at home was determined by weekly diary sheets completed by the participants.

### Analysis

Statistical analysis was carried out using IBM Statistical Package for Social Sciences (SPSS) for Windows, version 10. Baseline data in the two groups were summarised using descriptive statistics. Normality testing was undertaken using the Shapiro-Wilks test. If data was normally distributed the two groups were compared using an analysis of covariance (ANCOVA) adjusting for baseline scores. If the assumptions underpinning ANCOVA modelling were not met, a Mann Whitney test was carried out. Participants were analysed according to the group they were allocated. Only complete cases were analysed, and no imputation was undertaken. This reflects the fact that drop outs obtained were accounted for in the initial sample size calculation and there was no difference in the characteristics of those who dropped out at baseline.

## Results

**Fig. 3:**
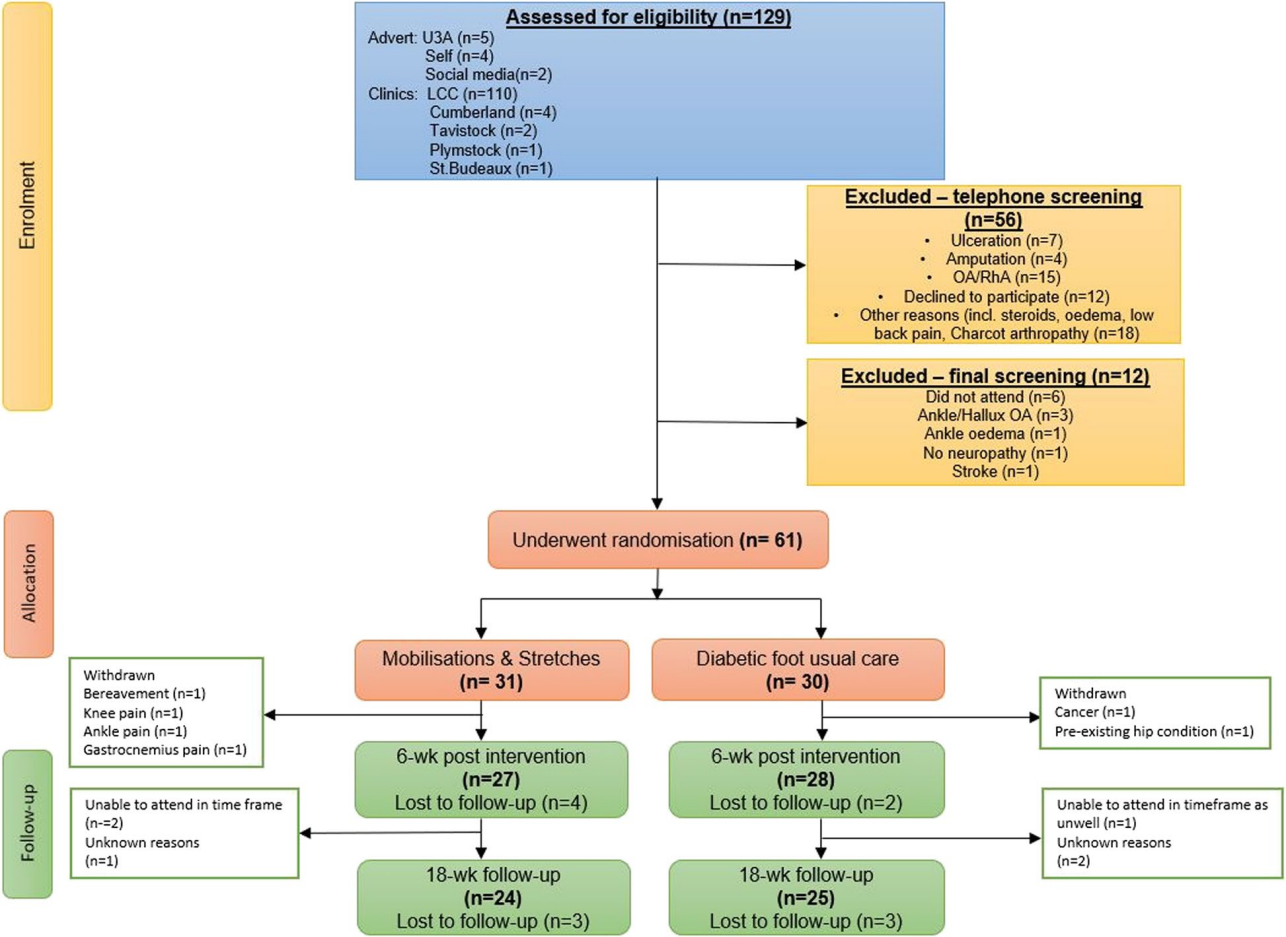
CONSORT flow diagram of the trial and study participants

Of the129 participants who volunteered for the study, 56 were excluded during telephone screening and a further 12 were excluded during the final screening. Reasons for exclusion are provided in the CONSORT diagram (Fig. 3). A total of 61 participants were randomly allocated (Intervention *n* = 31, Control *n* = 30). Six participants were lost to follow up at T1 and five at T2.

Baseline participant characteristics are presented in Table 2. There were no significant differences in any participant characteristics between groups.

**Table 2:** Participant characteristics

**Parameters**	**Baseline (All Participants)**			**Participants lost to follow-up**		**Remaining participants at post-intervention**		
	Intervention *N* = 31	Control *N* = 30	p	Intervention *N* = 4	Control *N* = 2	Intervention *N* = 27	Control *N* = 28	p
**Age (yrs)** ***Mean (std)***	70.49 (8.7)	68.3 (10.34)	t(59) = .89 *p* = .38	66.2 (10.56)	79.5 (4.94)	71.1 (8.45)	67.5 (10.20)	t(53) = -1.42 *p* = .31
**BMI (Kg/m**^**2**^**)** **Mean (std)**	32.67 (6.98)	31.9 (5)	U = 458.5 *p* = .93	30 (4.1)	29.1 (9.62)	33.1 (7.4)	32.1 (4.78)	U = 375.5 *p* = .97
**Neuropathy Severity** **(10 g Monofil No. correct responses)** **(Median score (IQR)**	4 (5.25)	3.5 (4)	U = 407.5 *p* = .40	1.5 (6)	3.5 (-)	4 (4)	3.5 (5)	U = 314.5, *p* = .28
**Neuropathy** **(Neurothesiometer)** **(Mean(std)**	32.10 (15)	33.7(14.6)	U = 447, *p* = .76	31.5(17)	33.3(16.6)	32.2(15.1)	33.8(14.82)	U = 359.5 *p* = .72
**Type I Diabetes** **No. (%)**	2(6.45)	5(16.66)	χ^2^(1) = .1.6 *p* = .21	0	0	2(7.69)	5(21.43)	χ^2^(3) = 2.34 *p* = .51
**Duration of Diabetes** **Mean (std)**	13.8 (9.75)	18.5 (13.3)	U = 385, *p* = .25	20.25 (18.7)	26 (4.24)	12.81 (7.9)	18 (13.6)	U = 319.5 *p* = .324
**Forefoot Deformity** **Mean (std)**	1.4 (.9)	1.4 (.8)	U = 458.5 *p* = .92	1.75(0.95)	0(0)	1.3 (0.9)	1.37 (.82)	U = 364.5 *p* = .81
**Peripheral Arterial Blood Supply** **Mean (std)**	2.5 (.67)	2.4 (.96)	U = 463, *p* = .98	2.75(0.5)	0(0)	2.5 (.69)	2.4 (.98)	U = 374.5 *p* = .95
**Limited Joint Mobility Syndrome** **Prayer Sign – no limitation** **No.(%)**	8(25.80)	11(36.66)	χ^2^(3) = 2.1 *p* = .54	2(40)	0(0)	6(23.07)	11(39.28)	χ^2^(9) = 2.5 *p* = .28
**Limited Joint Mobility Syndrome** **Table Top Sign – no limitation (%)**	9(29.03)	11(36.66)	χ^2^(3) = 2.80 *p* = .42	2(40)	0(0)	(7)26.92	11(39.28)	χ^2^(9) = 3.1 *p* = .22
**Foot Posture Index** **Mean (std)**	3.01 (3.87)	2.19 (4.05)	t(59) = .820 *p* = .42	2.4 (5.15)	2.5 (0.70)	3.11 (3.76)	2.16 (4.20)	t(53) = -.88 *p* = .26
**Callus forefoot Not Present** **No. (%)**	24(77.4)	22(73.3)	χ^2^(2) = .737 *p* = .69	3(75)	2(100%)	21(77.8)	20(71.4)	χ^2^(6) = 5.4 *p* = .49
**Callus hallux Not Present** **No. (%)**	27(87.1)	22(73.3)	χ^2^(2) = 1.83 *p* = .40	4(100)	1(50)	23(85.2)	21(75)	χ^2^(6) = 3.95 *p* = .68
**No. of Falls** **Median (IQR)**	0(1)	0 (0)	U = 378.5 *p* = .07	0 (0)	0(0)	0(1)	0 (0)	U = 294.5 *p* = .05

### Gait

#### Ankle dorsiflexion in gait

There was no difference in the primary outcome measure, maximal dorsiflexion during stance phase, between groups at the 6-week time point (T1) (*P* > 0.05) (Table 3) or at week-18 (T2) (*P* > 0.05) (Table 4) follow-up period.

**Table 3:** Walking related variables, sway and clinical data at baseline and 6 weeks follow up *p < 0.05, **p < 0.01

**Outcome**	**Control**			**Intervention**			**Statistical Testing**	**Hedges G (95% CI)** **Effect Size** **Hedges g** **(95% Confidence Interval)**
	n	Baseline Mean (SD)	6 weeks Follow up Mean (SD)	n	Baseline Mean (SD)	6 weeks follow up Mean (SD)		(95% CI)
Right Ankle DF stance (^o^)	28	20.5 (4.0)	20.2 (4.2)	27	20.4 (4.1)	21.3 (5)	*F* (1,52) = 1.4 *P* = .242	-0.31 (-0.22, 0.84)
Left Ankle DF stance(^o^)	28	21 (4.9)	21 (4.5)	27	20.7 (3.8)	21.9 (4.6)	*F* (1,52) = 0.96 *P* = .33	0.28 (-0.26, 0.81)
Right Ankle DF swing(^o^)	28	9 (4.1)	8.8 (3.5)	27	9.8 (3.5)	10.8 (4.5)	*F* (1,52) = 1.94 *P* = .169	0.30 (-0.23, 0.83)
Left Ankle DF swing(^o^)	28	9.7 (5)	10 (4.9)	27	8.9 (3.6)	10.6 (4.3)	*F* (1,52) = 1.01 *P* = .318	0.33 (-0.20, 0.86)
R Ankle total range(^o^)	28	24.3 (4.4)	24.8 (3.8)	27	23.9 (3.5)	24.6 (3.8)	*F* (1,52) = .015, *P* = .902	0.08 (-0.45, 0.61)
L Ankle total range(^o^)	28	24.8 (4.7)	25 (5)	27	24.3 (4.5)	25.4 (3.8)	*F* (1,52) = 1.04, *P* = .312	0.30 (-0.23, 0.83)
R Step length (mm)	28	1011.3 (149.6)	1070.4 (142.9)	27	978.6 (238.3)	1033.1 (288.4)	*F* (1,52) = .021, *P* = .884	-0.05 (-0.57, 0.48)
L Step length (mm)	28	994.3 (165)	1058.6 (150.6)	27	959.2 (232)	1035.6 (285.9)	*F* (1,52) = .082, *P* = .776	0.09 (-0.43, 0.62)
R Stride length (mm)	28	504.5 (76.9)	531.3 (701)	27	500 (126.4)	513.1 (146.3)	*F* (1,52) = .644, *P* = .426	-0.21 (-0.74, 0.32)
L Stride length (mm)	28	502.2 (88)	532.7 (78.2)	27	489 (124.9)	514 (144.1)	*F* (1,52) = .15, *P* = .703	-0.07 (-0.60, 0.46)
R Peak FF Pres. (KPa)	27	627 (216.3)	625 (202.2)	26	678.9 (257.5)	620.8 (189)	*F* (1,50) = .42, *P* = .519	-0.28 (-0.82, 0.26)
L Peak FF Pres. (KPa)	27	631.2 (221.8)	620.2 (194.8)	26	623.3 (198.3)	584.1 (157.5)	*F* (1,50) = .580, *P* = .450	-0.14 (-0.68, 0.40)
R FP ratio	27	1.6 (0.5)	1.5 (0.42)	26	1.5 (0.54)	1.4 (0.48)	*F* (1,50) = .006, *P* = .941	0.12 (-0.42, 0.65)
L FP ratio	27	1.53 (0.65)	1.39 (0.41)	26	1.30 (0.33)	1.33 (0.48)	U = 371, Z = –0.12, *P* = .906	0.43 (-0.11, 0.98)
Sway EO (mm/s)	28	21.5 (12.94)	20.54 (9.95)	27	22.65 (10.31)	20.49 (7.31)	U = 362, Z = -.035, *P* = .972	-0.20 (-0.73, 0.33)
Sway EC (mm/s)	28	40.31 (29.84)	36.38 (26.79)	27	41.20 (21.92)	34.50 (16.70)	U345, Z = -.329, *P* = .742	-0.18 (-0.71, 0.35)
R Static Ankle DF (cm)**	28	6.21 (4.18)	5.71 (3.70)	27	6.42 (2.90)	8.04 (3.19)	*F*(1,52) = 24.0 *P* = .000	1.25 (0.67, 1.82)
L Static Ankle DF (cm)**	28	6.64 (3.60)	6.36 (3.49)	27	7 (3.46)	8.52 (3.93)	*F*(1,52) = 13.03, *P* = .001	0.95 (0.39, 1.51)
R Static Hallux DF (cm)	28	17.44 (7.86)	16.93 (6.13)	26	12.82 (4.12)	15.64 (5.66)	*F*(1,51) = 1.446, *P* = .235	0.66 (0.11, 1.21)
L Static Hallux DF (cm)*	28	15.79 (8.15)	15.51 (7.1)	27	12.13 (6.50)	14.88 (6.59)	*F* = (1,52) = 4.071, *P* = .049	0.72 (0.17, 1.26)
Functional Reach (cm)*	28	27.61 (6.83)	27.89 (7.48)	27	25.90 (8.49)	29.03 (8.32)	*F*(1,52) = 5.674, *P* = .021	0.69 (0.14, 1.23)
Walking Speed (m/s)	28	12 (2.94)	11.62 (2.81)	27	12.5 (3.14)	11.41 (2.91)	U = 354.5, Z = -.165, *P* = .869	-0.11 (-0.62, 0.39)

*R* Right, *L* Left, *DF* Dorsiflexion, *Total Range* Difference between maximal dorsiflexion and maximal plantarflexion, *FF Pres* Forefoot Pressure, *FP ratio* Forefoot to rearfoot pressure ratio, *EO* Eyes open, *EC* Eyes closed, *SD* Standard deviation, *CI* Confidence interval

#### Stride length, forefoot PPP and F/R ratio

There were no differences in the gait-related secondary outcome measures between groups at T1 (Table 3) and T2 time points (Table 4).

**Table 4:** Walking related variables, sway and clinical data at baseline and 18 weeks follow up *p < 0.05, **p < 0.01

**Outcome**	**Control**			**Intervention**			**Statistical Testing**	**Hedges G (96% CI)** **Effect Size** **Hedges g** **(95% Confidence Interval)**
	n	Baseline Mean (SD)	18 weeks Follow up Mean (SD)	n	Baseline Mean (SD)	18 weeks follow up Mean (SD)		(95% CI)
Right Ankle DF stance (^o^)	24	20.35(4.2)	20.28 (4.5)	24	20.55(4.2)	19.92(4.98)	*F*(1,45) = .167, *p* = .685	-0.13 (-0.69, 0.44)
Left Ankle DF stance(^o^)	24	20.7(5)	20.8(4.6)21.4(5.7)	24	20.8(3.4)	21.4(5.7)	*F*(1,45) = .173, *P* = .679	0.10 (-0.46, 0.67)
Right Ankle DF swing(^o^)	24	8.88(4.1)	9.03(3.4)	24	9.84(3.7)	9.43(3.9)	*F*(1,45) = .002. *P* = .966	-0.12 (-0.68, 0.45)
Left Ankle DF swing(^o^)	24	9.9(5.3)	10.4(5.2)	24	9(3.7)	10.1(5.8)	U = 233, Z = -.915, *P* = .360	0.12 (-0.44, 0.69)
R Ankle total range(^o^)	24	24.62(4.51)	24.2(3.5)	24	24.05(3.6)	23.7(4.2)	*F*(1,45) = .029, *P* = .865	0.01 (-0.55, 0.58)
L Ankle total range(^o^)	24	25.1(5)	24.8(5.2)	24	24.1(4.5)	25.4(4.2)	*F*(1,45) = 1.302, *P* = .260	0.39 (-0.18, 0.96)
R Step length (mm)	24	1013.3(150)	1058.3(134.9)	24	975.3(248.5)	1017.5(291)	U = 253, Z = -.489, *P* = .625	-0.03 (-0.59, 0.54)
L Step length (mm)	24	996.6(166.2)	1050.8(126.4)	24	954.3(242)	982.8(275)	U = 244.5, Z = -.670, *P* = .503	-0.24 (-0.81, 0.33)
R Stride length (mm)	24	506.6(75.3)	523(63.8)	24	498.8(132.1)	504.1(152.2)	U = 252, Z = -.511, *P* = .610	-0.20, (-0.77, 0.36)
L Stride length (mm)	24	502.2(88.2)	512.6(63.4)	24	486.6(130.9)	499.7(154.3)	*F*(1,45) = .008, *P* = .930	0.05 (-0.52, 0.61)
R Peak FF Pres. (KPa)	24	639.9(209.2)	572.6(157.5)	23	683.9(271.5)	576.9(232.8)	*F*(1,44) = .089, *P* = .767	-0.18 (-0.76, 0.39)
L Peak FF Pres. (KPa)	24	633.2(215.2)	582(156.2)	23	629.7(206.5)	552.8(188.6)	*F*(1,44) = .340, *P* = .563	-0.11 (-0.69, 0.46)
R FP ratio	24	1.6(0.5)	1.6(0.5)	23	1.5(0.6)	1.5(0.5)	*F*(1,44) = .029, *P* = .866	0.06 (-0.51, 0.63)
L FP ratio	24	1.5(0.7)	1.5(0.7)	23	1.3(0.4)	1.4(0.3)	U = 282, Z = -.114, *P* = .910	0.16 (-0.41, 0.73)
Sway EO (mm/s)	24	20.1(9.8)	17.5(6.4)	24	22.8(10.6)	21.6(9.4)	U = 236, Z = -.851, *P* = .395	0.22 (-0.35, 0.78)
Sway EC (mm/s)	24	38.8(26.3)	32.8(20.1)	24	41.2(22.5)	37.5(20.3)	U = 239, Z = -.787, *P* = .431	0.16 (-0.40, 0.73)
R Static Ankle DF (cm)**	25	6.5 (4.3)	6.2(3.8)	24	6.3(3)	9(3.3)	*F*(1,46) = 31.346, *P* = 0.000	1.54 (0.90, 2.17)
L Static Ankle DF (cm)**	25	7.2(3.3)	6.7(3.2)	24	6.6(3.6)	9.5(3.7)	U = 170.5, Z = -2.43, *P* = .015	1.43 (0.80, 2.05)
R Static Hallux DF(^o^)**	25	18.1(8)	16.6(6.2)	23	12.5(4.2)	17.4(5)	*F*(1,45) = 14.833, *P* = 0.000	1.51 (0.86, 2.15)
L Static Hallux DF(^o^)	25	16(8.6)	14.4(5.7)	24	11.6(6.1)	16.8(5.4)	U = 226.5, Z = -1.26, *P* = .208	1.10 (0.50, 1.70)
Functional Reach (cm)*	25	27.3(7.1)	27.6(7.8)	24	25.3(8.5)	29.2(9.4)	*F*(1,45) = 7.063, *P* = .011	0.81 (0.22, 1.40)
Walking Speed (m/s)	25	12(2.9)	11.7(2.8)	24	13.1(4.2)	11.3(3.7)	U = 243, Z = -.918, *P* = .358	-0.51 (-1.09, 0.06)

*R* Right, *L* Left, *DF* Dorsiflexion, *Total Range* Difference between maximal dorsiflexion and maximal plantarflexion, *FF Pres* Forefoot Pressure, *FP ratio* Forefoot to rearfoot pressure ratio, *EO* Eyes open, *EC* Eyes closed, *SD* Standard deviation, *CI* Confidence interval

#### Balance

There were no differences between groups in postural sway measurements with eyes open and eyes closed at T1 (Table 3) and T2 (Table 4). However, differences between groups in FRT were observed. At T1 (*P* = .021), functional reach increased in the intervention group increased by 3.1 cm (± 4.4) compared to 0.3 cm (± 3.9) in the control group (Table 3). At T2 (*P* = .011), the intervention group increased by 3.9cms (± 4.3) and by 0.3 cm (± 4.3) in the control group (Table 4).

#### Bilateral static ankle joint dorsiflexion

At T1, there was an increase in left and right static ankle dorsiflexion in the intervention group compared to the control group. Right static ankle dorsiflexion increased in the intervention group by 1.62 cm (± 1.9) and decreased by -0.50 cm (± 1.4) in the control group. Left ankle static dorsiflexion increased in the intervention group by 1.52 (± 2.3) cm and decreased by -0.3 cm (± 1.4) in the control group (Table 3).

At T2, right static ankle dorsiflexion remained increased in the intervention group compared to the control group (*p* < 0.05). Between baseline and T2 follow up right ankle dorsiflexion increased by 2.7 cm (± 2.1) in the intervention group and decreased by -0.3 cm (± 1.8) in the control group. The left static dorsiflexion was not normally distributed at T2 in the control group. Therefore, the post intervention measures were compared using a Mann Whitney test. Left static ankle dorsiflexion was statistically significant (*P* = .015) at T2. Between baseline and T2 follow up left ankle dorsiflexion increased by 2.9 cm (± 3.1) in the intervention group and decreased by 0.3 cm (± 3.1) in the control group (Table 4).

#### Bilateral static hallux dorsiflexion

At T1, there was an increase in static left hallux dorsiflexion in the intervention group compared to the control group. Left hallux dorsiflexion increased by 2.8° (± 3.1) in the intervention group and decreased by -0.3° (± 5.0) in the control group (Table 3).

At T2, the right static hallux dorsiflexion range was higher in the intervention group at 18 weeks. Between baseline and 18 weeks follow up right static hallux dorsiflexion increased by 5.2° (± 4.0) in the intervention group and decreased by -1.6° (± 5.3) in the control group (Table 4).

#### Adherence rates (Additional file 4)

The percentage of people who returned diaries in the intervention group (equ 1) was 96% (30 out of 31). The percentage of weekly diaries returned in the intervention group (equ 2) was 77.6% (range 0–100%).The main cause of data loss was due to the non-return of the week 6 diary. Diaries for weeks 1–5 were returned to the treating therapist at the face-to-face intervention session. In contrast, the last diary sheet was returned after the last intervention session and was meant to be returned to the school’s administrator team when the participant attended the T6 appointment. Participants ticked a box to indicate if they had undertaken the prescribed exercises. The frequency of self-reported exercise was taken as a measure of compliance. Average percentage exercise compliance (equ 4) of the returned diaries was 83.37% (range 0.4–114.3%). Therapist notes indicated that the mobilisation protocol was adhered to in 100% of cases.

#### Adverse events (AEs)

There were 12 AEs (1 in the control group, 11 in the intervention group in nine participants). Six participants in the intervention group presented with musculoskeletal symptoms including bruising to the plantar aspect of the big toe, soreness/pain to the gastrocnemius muscle or ankle/knee joint. Two participants withdrew due to pain. In one this could be related to aggravating a pre-existing injury secondary to a road traffic accident. In the other participant their knee pain resolved after stopping the exercises, but they decided to withdraw. In all other participants their pain resolved following the physiotherapist’s advice and they completed the home-based stretches / mobilisation program. No foot ulcers were reported in either group.

## Discussion

This study compared the combined effects of a 6-week intervention of foot and ankle mobilisations with home-based stretches, to usual care in people with DPN and LJMS on ankle and big toe DF ROM, PPPs and balance.

### Changes in static ankle and 1^st^ MTPJ DF ROM, functional reach

In our literature search, we have not identified any study investigating the effect of mobilisations combined with stretches on ankle ROM in people at risk of diabetic foot ulcer [32]. Following 6-weeks of the intervention programme, there was an increase of 1.6 cm in the right ankle dorsiflexion and 1.5 cm in the left ankle dorsiflexion between groups as assessed using the lunge test. The literature suggests that every 1 cm distance away from the wall equates to approximately 3.6° of ankle DF ROM [69]. Based on this formula, our intervention increased right ankle DF ROM by approximately 5.8° and for the left ankle by approximately 5.5°. Our findings support those from studies who employed the same mobilisation component but recruited from a population with chronic ankle instability. These findings suggest that mobilisations may improve an acquired limitation of the ankle joint ROM. In this prior work, mobilisation was either delivered for a shorter duration and with no stretches [30]; or similarly combined mobilisations and stretches but were delivered at a smaller intervention dosage [31].

A recent study investigated the effects of gastro-soleus stretches in people with diabetes and LJMS [70]. Their findings did not support the use of stretches for increasing static ankle DF ROM (+ 1.3°, 95% CI: -0.3 to 2.9, *p* = .101). Further a Cochrane review [71] on the effects of stretches for the treatment and prevention of contractures in people with neurological conditions reiterates an absence of effect of stretches on joint mobility. However, neither study used mobilisations as part of their intervention and Harvey et al. based their review on a different population to the one assessed in this study. On this basis, we suggest that stretches alone may not be enough to increase ankle ROM in people with diabetes.

Our study observed between group improvements in FRT, a measure of balance. The findings concur with the subjective reports of improved balance confidence obtained by our own embedded qualitative study [72]. Moreover, these results agree with studies employing a similar mobilisation intervention in people with chronic ankle instability who measured standing balance with the Star Excursion Balance Test (SEBT) [30, 49]. SEBT test shares a forward reach component like the FRT, however due to the differences in the study population and the outcome measure, direct comparisons cannot be drawn. The improvement in FRT could reflect the improvements in ankle ROM. There are several ways in which people can undertake the FRT, either by rotating about their ankles and/or about their hips or trunk [73, 74]. This does require sufficient strength and balance as well as ROM.

### Changes in dynamic ankle DF ROM, PPPs and postural sway

Contrary to our hypothesis, our study found no group differences in ankle and 1^st^ MTPJ DF ROM, PPPs and postural sway following the 6-week intervention period. A causative link between limited ankle DF ROM and elevated PPPs has been suggested and further evidenced by studies where the surgical elongation of the Achilles tendon has been associated with a reduction in forefoot PPPs [20, 22, 75]. Contrary to this hypothesis, in our study the increase in static ankle joint dorsiflexion ROM was not accompanied by increase in dynamic ankle dorsiflexion or a reduction in PPP. Our findings therefore suggest that ankle and 1^st^ MTPJ mobilisations increase have no effect on PPP and will not reduce foot ulcer risk in people with diabetes and neuropathy. There are several possible explanations for this finding. Inclusion criteria for participants with LJMS recruited with ankle stiffness was defined as 0° or less of ankle DF measured with a static, non-WB ankle dorsiflexion test in long sitting [76]. However, the same participants exhibited mean dynamic baseline ankle DF range of 20.4° to 21° in stance phase while walking. It maybe that there was no functional requirement for these participants to “use” the increased range seen with mobilisation, or that the single segment biomechanical foot model used to calculate dynamic range of ankle DF in stance phase failed to isolate the ankle joint and instead captured the accumulative range of motion occurring within the ankle and midfoot.

Lastly, no significant changes were noted in postural sway between intervention and control. Measures of balance were in part assessed to determine if the intervention led to a reduction in balance. It was hypothesised that a reduction in ankle stiffness generated by intrinsic torque [77] may lead to an increase in sway [78]. The results highlight that no reduction in balance was seen following the intervention, however this could be attributed to the small effect of the intervention an ankle ROM.

### Limitations

Our study had several limitations. As there are two components to the intervention, it is not possible with the current study design to ascertain which of these were effective in improving static ankle and hallux ROM or whether their effectiveness arises from an interaction of the two. However, the rationale for combining the two interventions is that it reflects usual musculoskeletal practice [79, 80] and it might have improved adherence rates of the participants to the stretches. Another limitation was the method used to define ankle stiffness and the means of capturing dynamic ankle ROM (i.e., single vs multi-segmental foot model). The follow-up period was only 18 weeks therefore we were unable to determine if any changes or high adhesion rates were maintained over time. Finally, this PoC study was not large enough or followed up long enough to use ulceration occurrence as a primary outcome measure. However, a strength of the study was that it recruited to a full sample, and no major adverse effects (i.e., ulceration, Charcot arthropathy, falls) were reported in the intervention group.

### Future directions

There is an absence of existing evidence investigating the effect of mobilisations in people with diabetes and neuropathy. Whilst the result of this study suggests that mobilisations may hold potential for increasing static ankle and first MTPJ ROM, no functional or objective clinical benefits were established. Further randomised control trials are therefore needed to evaluate the effect of mobilisations and stretches on people with diabetes and neuropathy. This is in line with recent recommendations made by a systematic review which advocated the use of foot and ankle exercise programmes but suggested further focus on the effects of specific components of these programmes [40].

Future research is needed to address issues on study design and implementation. Firstly, the inclusion criteria need to be refined to confirm LJMS and the presence of a functional ankle equinus. Secondly, a factorial study design might be more appropriate to separate the effectiveness between mobilisations and stretches. In future trials, more attention is needed to describe the characteristics of care provided to participants in the control group and to those of the intervention group and whether usual care varied between groups which can be viewed as a potential confounder [81]. Continuing to monitor and report exercise adherence during the follow-up period is also important to enhance our understanding of long-term adherence rates. More work is needed to increase our understanding into the relationship between ankle measurements and forefoot PPPs.

In addition, the choice of outcome measures (OMs) need to be re-evaluated and consideration needs to be given to OMs that are more meaningful to participants such as using patient-reported outcome measures (foot and ankle disability index, EQ5DL) and clinical outcome measures of foot health including incidence of ulceration. Lastly, the exploring the acceptability and feasibility of delivering the intervention will also be an important component of a future study.

## Conclusions

This is the first study to propose that ankle and 1^st^ MTPJ mobilisations together with home-based stretches are effective at increasing static measures of joint range of motion, but not reducing PPP in people at risk of diabetic foot ulceration. Whilst the intervention showed some potential for improving ankle, hallux joint mobility and anteroposterior stability limits in people with diabetes and neuropathy, no functional benefit or measurable reduction in ulcer risk was observed. It also raised the question whether mobilisations and home-based stretches have a role to play in a select sub-group amongst people with DPN, suggesting a refined inclusion criteria list for ankle equinus. Overall and in line with the recommendations made by IWGDF [40, 82], further research is needed to establish the positive effects of physical therapy in improving foot health related outcomes in people with diabetes.

### Supplementary Information

**Additional file 1.** Home Exercise Programme.

**Additional file 2.** Participant Exercise Diary Sample.

**Additional file 3.** Physiotherapy treatment proforma sheet.

**Additional file 4.** Exercise adherence – explanation of equations.

## Data Availability

All anonymised data can be made available from the corresponding author upon reasonable request.

## Abbreviations

AEs: Adverse Events
ANCOVA: Analysis of Covariance
CONSORT: Consolidated Standards of Reporting Trials
CoM: Centre of Mass
DF: Dorsiflexion
DFU: Diabetic Foot Ulceration
DM: Diabetes Mellitus
DPN: Diabetic Peripheral Neuropathy
EQ5DL: EuroQol instrument 5 Dimensions 5 Levels
FPI: Foot Posture Index
FRT: Functional Reach Test
F/R ratio: Forefoot-to-Rearfoot Pressure ratio
ICC: Interclass correlation coefficient
IWGDF: International Working Group on the Diabetic Foot
KGs: Kilograms
LJMS: Limited Joint Mobility Syndrome
MATLAB: Matrix Laboratory
MTP: Metatarsophalangeal
OMs: Outcome Measures
PoC: Proof-of-concept
PPPs: Peak Plantar Pressures
RCT: Randomised Controlled Trial
REC: Research Ethics Committee
ROM: Range of Motion
SEBT: Star Excursion Balance Test
SPSS: Statistical Package for Social Sciences
WB: Weight Bearing
