# Supplementary material for: Effects of foot and ankle mobilisations combined with home stretches in people with diabetic peripheral neuropathy: a proof-of-concept RCT
Source: J Foot Ankle Res. 2023 Dec 6;16:88. doi: 10.1186/s13047-023-00690-4 (PMC10699018; doi:10.1186/s13047-023-00690-4)
Supplement: Supplementary file 1 — Additional file 1. Home Exercise Programme. [file 13047_2023_690_MOESM1_ESM.docx]

**Additional File 1: Home Exercise Programme**

Dear

**Can a form of physiotherapy reduce stiffness in the foot joints of people with diabetes?**

This leaflet provides written explanation and should act as a reminder of the exercises prescribed to you by your physiotherapist. These exercises should **NOT** cause you any pain but you should feel a sensation of “pulling”, “stretching” or “tightness” in the area that you are stretching. If any of these exercises cause you pain or discomfort you should stop them immediately and inform your physiotherapist at your next appointment.

**Calf stretches**

Standing with the leg to be stretched at the back and both hands against a wall at shoulder height.

**EXERCISE 1**: Bending the front leg at knee level and leaning forwards, keeping the back leg and knee straight and pushing the heel down into the floor; 20-30 sec hold, 2 times each leg twice daily.


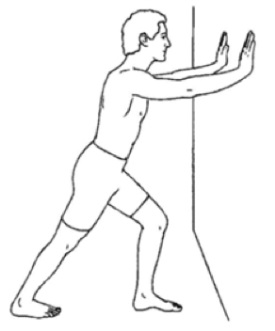


**EXERCISE 2**: Bending both legs at knee level and pushing the heel of the back leg down into the floor; 20-30 sec hold, 2 times each leg twice daily.


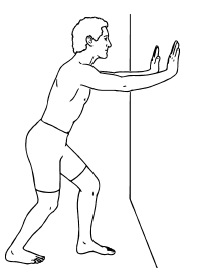


**Plantar fascia stretch**

**EXERCISE 3**: Place your toes, concentrating on your big toe, against a wall and lean forward or push your knee forward over your toes; 20-30 hold, 2 times each leg twice daily.


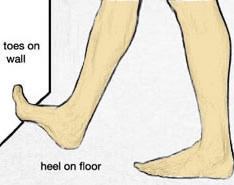


If you feel confused on how to carry out any of the exercises or you would like further explanation, please let your physiotherapist know who will be happy to demonstrate to you how to perform these exercises efficiently and safely.

Please record the exercises by ticking them as appropriately in your exercise diary sheet supplied to you.

**What if I have any further questions or require further information?**

Contact:

Jon Marsden

01752 587 590

jonathan.marsden@plymouth.ac.uk (Mon Friday)

Alec Rickard

01752 588850

[alec.rickard@plymouth.ac.uk](mailto:alec.rickard@plymouth.ac.uk)

*Thank you for taking part in the study*
