# Supplementary material for: Effects of foot and ankle mobilisations combined with home stretches in people with diabetic peripheral neuropathy: a proof-of-concept RCT
Source: J Foot Ankle Res. 2023 Dec 6;16:88. doi: 10.1186/s13047-023-00690-4 (PMC10699018; doi:10.1186/s13047-023-00690-4)
Supplement: Supplementary file 3 — Additional file 3. Physiotherapy treatment proforma sheet. [file 13047_2023_690_MOESM3_ESM.docx]

**Additional file 3: Physiotherapy treatment proforma sheet**

**Date:**

**Session number**:

| Reported issues following last treatment (soreness, pain, swelling, redness): |
| --- |

*(if none reported please put NONE in the box above)*

**Treatment dosage (as per treatment protocol)**

**Ankle traction**

**R/Ankle**: 2 x 2-min sets of traction (1-min rest btw sets) Completed Yes □ No □

| Reasons for no completion:  Dosage delivered: | **R** |
| --- | --- |

**L/Ankle**: 2 x 2-min sets of traction (1-min rest btw sets) Completed Yes □ No □

| Reasons for no completion:  Dosage delivered: | **L** |
| --- | --- |

**Big toe traction**

**R/1^st^ MTPJ**: 2 x 1 min sets of traction (30-sec rest btw sets) Completed Yes □ No □

| Reasons for no completion:  Dosage delivered: | **R** |
| --- | --- |

**L/1^st^ MTPJ**: 2 x 1 min sets of traction (30-sec rest btw sets) Completed Yes □ No □

| Reasons for no completion:  Dosage delivered: | **L** |
| --- | --- |

**Ankle Gd III AP mobs**

**R/Ankle**: 4 x 2 mins sets of mobs (1-min rest btw sets) Completed Yes □ No □

| Reasons for no completion:  Dosage delivered: | **R** |
| --- | --- |

**L/Ankle**: 4 x 2 mins sets of mobs (1-min rest btw sets) Completed Yes □ No □

| Reasons for no completion:  Dosage delivered: | **L** |
| --- | --- |

**Big toe Gd III PA mobs**

**R/1^st^ MTPJ**: 2 x 2 mins sets of mobs (1-min rest btw sets) Completed Yes □ No □

| Reasons for no completion:  Dosage delivered: | **R** |
| --- | --- |

**L/1^st^ MTPJ**: 2 x 2 mins sets of mobs (1-min rest btw sets) Completed Yes □ No □

| Reasons for no completion:  Dosage delivered: | **L** |
| --- | --- |

| Additional treatment notes: |
| --- |

**Home exercise programme**

Weekly Exercise sheet checked and completed (handed-in): Yes □ No □

| If no, please state action taken: |
| --- |

Exercises visually checked (demo by participant): Yes □ No □

Advise required? Yes □ No □

Demo exercises (by therapist): Yes □ No □

| Treatment completed by: | Date: |
| --- | --- |
