# Supplementary material for: Effects of foot and ankle mobilisations combined with home stretches in people with diabetic peripheral neuropathy: a proof-of-concept RCT
Source: J Foot Ankle Res. 2023 Dec 6;16:88. doi: 10.1186/s13047-023-00690-4 (PMC10699018; doi:10.1186/s13047-023-00690-4)
Supplement: Supplementary file 4 — Additional file 4. Exercise adherence – explanation of equations. [file 13047_2023_690_MOESM4_ESM.docx]

**Additional file 4: Exercise adherence – explanation of equations**

Adherence was assessed via the diaries by determining:

1. **Percentage of people who returned diaries as defined by**:

No. people retuning a diary in the intervention group x 100 (equ 1)

Number of participants in the intervention group

1. **Percentage of weekly diaries returned as defined by**:

Percentage diary returns per person*___________ (equ 2)

31 (total number of participant in intervention group)

*Where percentage diary returns =

No. of diaries returned per person_____ x100 (equ 3)

6 (total number of possible diary returns)

1. **Average percentage exercise compliance as defined by**:

Percentage exercise compliance* _____________ (equ 4)

31 (total number of participant in intervention group)

*Where percentage exercise compliance=

No. exercise sessions ticked x100 (equ 5)

No. diaries returned x 42

where 42 indicates the total number of prescribed sessions per week (7 days x 2 sessions / day x3 exercise per session). If people indicated that they undertook more than two sessions in a day these were counted and it is therefore possible to have over 100% compliance using this measure.
